# Safety, efficacy, and opioid-sparing effects of ketamine for analgesia in traumatic chest injury and rib fractures: a systematic review and meta-analysis

**DOI:** 10.3389/fmed.2026.1914190

**Published:** 2026-09-29

**Authors:** Kaiyao Zhang, Jiangbo Zheng, Yafeng Zhang, Xiaoming Xia, Yuqing Kang, Renzhong Shi

**Affiliations:** 1 Department of Thoracic Surgery, Jinshan Branch of Shanghai Sixth People’s Hospital, Shanghai, China; 2 Department of Anaesthesiology, Jinshan Branch of Shanghai Sixth People’s Hospital, Shanghai, China

**Keywords:** analgesia, ketamine, length of stay, morphine equivalents, opioid-sparing analgesia, pain management, respiratory insufficiency, rib fractures

## Abstract

**Background:**

Traumatic chest injury and rib fractures are frequently associated with severe pain, impaired ventilation, opioid exposure, and pulmonary complications. Ketamine has been proposed as an opioid-sparing analgesic adjunct, but its efficacy and safety in this population remain uncertain.

**Objective:**

To evaluate the efficacy, opioid-sparing effect, and safety of ketamine for analgesia in patients with traumatic chest injury and/or rib fractures.

**Methods:**

We systematically searched five databases for studies evaluating ketamine in traumatic chest injury or rib-fracture populations. Eligible designs included randomized trials, observational comparative studies, case series, and conference abstracts. Outcomes included pain scores, opioid consumption, hospital length of stay, epidural placement, respiratory failure, mortality, sedation, and hallucinations/delirium/agitation. Random-effects meta-analysis was performed when sufficient comparative data were available. Risk of bias and certainty of evidence were assessed using design-appropriate tools and GRADE.

**Results:**

Eleven studies were included in the systematic review, of which seven contributed to quantitative synthesis. Ketamine did not significantly reduce pain at 12–24 h (MD, −0.10 points; 95% CI, −0.69 to 0.50; *p* = 0.75) or 24–48 h (MD, 0.24 points; 95% CI, −0.64 to 1.12; *p* = 0.59). Day-1 opioid consumption showed a non-significant reduction with ketamine (MD, −9.55 morphine-equivalent units; 95% CI, −20.61 to 1.52; *p* = 0.09), while total opioid consumption was not significantly different (MD, −9.35; 95% CI, −25.42 to 6.73; *p* = 0.25). No statistically significant differences were detected for hallucinations/delirium/agitation (RR, 1.65; 95% CI, 0.83 to 3.29; *p* = 0.15) or respiratory failure (RR, 0.79; 95% CI, 0.20 to 3.10; *p* = 0.73). In an exploratory subgroup of patients with ISS ≥15, opioid consumption was lower at 12–24 h (MD, −37.39 mg; 95% CI, −73.98 to −0.81; *p* = 0.05), but the subgroup interaction was not significant (*p* = 0.28).

**Conclusion:**

Current evidence does not support routine ketamine use for patients with traumatic chest injury or rib fractures. Whether ketamine provides an opioid-sparing benefit in selected patients with greater injury severity remains uncertain, and the available subgroup finding should be considered exploratory pending confirmation in adequately powered randomized trials.

**Systematic Review Registration:**

PROSPERO, identifier CRD420261395009.

## Introduction

Traumatic chest injury is a common and clinically important component of blunt trauma, with rib fractures representing one of its most frequent and painful manifestations. Rib fractures are not merely radiographic findings; they are associated with impaired ventilation, reduced cough effectiveness, atelectasis, pneumonia, respiratory failure, prolonged hospitalization, and increased mortality, particularly in older adults and patients with multiple fractures or greater injury severity (1, 2). The pathophysiologic link between pain and pulmonary deterioration is central to management; patients with inadequately controlled chest wall pain often avoid deep inspiration, coughing, and mobilization, thereby increasing the risk of retained secretions and secondary pulmonary complications (1, 3).

Effective analgesia is therefore a cornerstone of nonoperative management for traumatic rib fractures and blunt chest trauma. Contemporary care commonly relies on multimodal analgesia, including acetaminophen, nonsteroidal anti-inflammatory drugs (NSAIDs), systemic opioids, regional anesthesia, pulmonary hygiene, and early mobilization (2, 4). However, each analgesic strategy has limitations. Opioids remain widely used because of their rapid and reliable analgesic effects, but their adverse effects, including sedation, nausea, ileus, delirium, respiratory depression, and risk of persistent opioid exposure, are particularly concerning in patients with chest trauma, where preservation of ventilation is essential (4, 5). Regional techniques such as thoracic epidural, paravertebral, erector spinae plane, and serratus anterior plane blocks may provide effective analgesia, but their use can be limited by anticoagulation, hypotension, spinal injury, technical availability, patient positioning, or institutional expertise (3, 4). Ketamine has emerged as a potentially attractive adjunct in this setting because it provides analgesia primarily through N-methyl-D-aspartate receptor antagonism and may reduce central sensitization and opioid requirements at subanesthetic doses (6). Consensus guidance supports the use of subanesthetic intravenous ketamine for acute pain in selected perioperative, emergency, and opioid-tolerant populations, although dosing, monitoring, and patient selection remain important safety considerations (7). In patients with traumatic chest injury, the theoretical advantages of ketamine include opioid-sparing analgesia, preservation of respiratory drive, and hemodynamic stability. At the same time, concerns remain regarding psychotomimetic effects, agitation, sedation, hypertension, nausea, and delirium, especially in older or critically ill patients.

Clinical evidence for ketamine in traumatic rib fractures and chest injury has expanded but remains inconsistent. Early observational evidence suggested that low-dose ketamine may reduce pain scores and opioid consumption in adult trauma intensive care unit patients with rib fractures (8). Subsequent randomized trials in adults and older patients with multiple rib fractures found no clear overall improvement in pain scores, although subgroup signals suggested possible opioid-sparing effects among more severely injured patients (9, 10). More recent trials have produced mixed findings; one placebo-controlled trial found no reduction in morphine milligram equivalents or pulmonary complications when low-dose ketamine was added to an evidence-based rib-fracture protocol (11), whereas studies in broader chest-trauma or trauma populations have evaluated ketamine against active comparators or as part of pragmatic multimodal regimens (12, 13). These differences in design, population, ketamine dose, comparator, outcome timing, and injury severity complicate interpretation.

Existing reviews and guidelines have addressed analgesia for rib fractures broadly, but the ketamine-specific evidence base has not been fully clarified across traumatic chest injury and rib-fracture populations (2, 4). A focused synthesis is therefore needed to determine whether ketamine improves pain control, reduces opioid exposure, affects hospital length of stay or procedure use, and whether these potential benefits are offset by neuropsychiatric, respiratory, or mortality-related safety signals. We conducted this systematic review and meta-analysis to evaluate the efficacy, opioid-sparing effect, and safety of ketamine for analgesia in patients with traumatic chest injury and/or rib fractures.

## Methods

### Literature search and study selection

This systematic review and meta-analysis was conducted and reported in accordance with the Preferred Reporting Items for Systematic Reviews and Meta-Analyses 2020 statement (14). The protocol was also pre-registered into PROSPERO (CRD420261395009).

A comprehensive literature search was performed in PubMed, Web of Science, Scopus, Embase, and Cochrane CENTRAL from database inception to August 2026. The search strategy combined terms related to ketamine with terms related to traumatic chest injury and rib fractures. Search terms included “ketamine,” “Ketalar,” “CI 581,” “ketamine hydrochloride,” “rib fracture,” “chest injury,” and “thoracic injury,” with database-specific adaptations. Detailed keywords used for search are presented in Supplementary Table S1. The complete search strings for each database are also provided in Supplementary Table S2. Additional records were identified through citation searching of relevant studies and reviews.

All retrieved records were imported into EndNote X8, and duplicates were removed before screening. Two reviewers (KZ, JZ) independently screened titles and abstracts for potential eligibility. Full texts were then assessed against predefined eligibility criteria. Disagreements were resolved through discussion, and when necessary by consultation with a third reviewer (RS). The study-selection process was summarized using a PRISMA flow diagram. Conference abstracts were considered eligible when sufficient information was available to determine population, intervention, comparator, and outcomes; however, abstracts with insufficient quantitative data were included only in the narrative synthesis.

### Inclusion criteria

Studies were eligible if they met the following criteria: (1) included patients with traumatic chest injury, traumatic rib fractures, blunt thoracic trauma, or closely related chest-wall trauma populations; (2) evaluated ketamine administered for analgesia, opioid-sparing effect, sedation-assisted analgesia, or pain control, either as a bolus, continuous infusion, or adjunct to multimodal analgesia; (3) included a comparator group such as placebo, standard care, usual multimodal analgesia, or an active analgesic comparator, or provided clinically relevant uncontrolled data for narrative synthesis; and (4) reported at least one relevant efficacy, opioid-use, procedural, length-of-stay, respiratory, or safety outcome.

For terminological consistency, “traumatic chest injury” is used throughout this review as the overarching term encompassing acute traumatic injuries of the chest. “Rib fractures” refers specifically to populations with identified traumatic rib fractures, while “blunt chest trauma” is used only when the original study explicitly restricted eligibility to a blunt mechanism of injury.

Eligible study designs included randomized controlled trials, prospective or retrospective cohort studies, matched case-control studies, case series, and conference abstracts. Studies were excluded if they were reviews, editorials, animal or laboratory studies, non-traumatic populations, postoperative thoracic surgery populations without traumatic injury, studies not involving ketamine, studies not involving traumatic chest injury or rib fractures, or studies without extractable clinical outcomes. For quantitative synthesis, studies were required to provide comparative data with sufficient numerical information to calculate an effect estimate. Single-arm studies, case series, studies with overlapping populations, and studies with insufficient quantitative data were summarized narratively unless a unique extractable outcome was available.

### Data extraction

Two reviewers (KZ and JZ) independently extracted data using a standardized extraction form. Extracted variables included first author, publication year, country, study design, clinical setting, population characteristics, inclusion and exclusion criteria, sample size, age, sex, injury severity, number of rib fractures, ketamine dose, route, timing, duration, comparator intervention, co-interventions, and follow-up duration. Outcomes extracted included pain scores, numeric pain score area under the curve (NPS AUC), visual analog scale (VAS) or numeric rating scale values (NRS), opioid consumption reported as morphine milligram equivalents (MME) or oral morphine equivalents (OME), hospital length of stay (LOS), intensive care unit (ICU) length of stay, respiratory failure or insufficiency, pulmonary complications, mortality, sedation, hallucinations, delirium, agitation, other adverse events, and procedural outcomes such as epidural placement.

Opioid consumption was extracted using the morphine-equivalent metric reported by each study, including MME or OME. When consumption was reported as an hourly morphine-equivalent rate, the mean and corresponding measure of dispersion were multiplied by 24 to express consumption in milligrams per day. Studies already reporting milligrams per day required no time-unit conversion, and no universal conversion between aggregate OME and MME values was applied. For continuous outcomes, means and standard deviations were extracted when available. When outcomes were reported as medians with interquartile ranges (IQRs) or ranges, means and standard deviations (SDs) were estimated using established methods for meta-analysis when appropriate (15–17) If data were reported only descriptively, or without adequate dispersion, the outcome was not pooled unless reliable extraction was possible. When multiple publications or abstracts derived from the same parent cohort, overlapping outcomes were not double-counted; only unique outcomes were included in the relevant synthesis.

Consistent with the prespecified PROSPERO registration, the review included two co-primary efficacy outcomes: pain intensity and opioid consumption. Pain was evaluated using NPS AUC, at the reported assessment times, while opioid consumption was evaluated using study-reported morphine-equivalent measures. A separate prespecified safety outcome domain included hallucinations, delirium, agitation, sedation, respiratory failure or respiratory insufficiency, and mortality. Secondary outcomes included hospital and intensive care unit length of stay, pulmonary complications, epidural placement, and other procedural analgesic interventions. Each outcome was synthesized and assessed separately; no composite efficacy–safety endpoint was used.

### Quality assessment

Risk of bias was assessed independently by two reviewers (YZ, and XX). Randomized controlled trials were evaluated using the revised Cochrane Risk of Bias tool for randomized trials, version 2 (RoB2), which assesses bias arising from the randomization process, deviations from intended interventions, missing outcome data, outcome measurement, and selection of the reported result (18). Non-randomized comparative studies were assessed using ROBINS-I, which evaluates bias due to confounding, participant selection, intervention classification, deviations from intended interventions, missing data, outcome measurement, and selection of reported results (19). Any disagreements were resolved by consensus (RS). Conference abstracts, uncontrolled case series, and single-arm registry reports were not treated as equivalent to fully reported comparative trials. These studies were appraised narratively using the JBI critical appraisal tool (20) with attention to study design, completeness of reporting, outcome ascertainment, lack of control group, sample size, and availability of extractable data. Studies with major limitations in design, reporting, or extractability were included in the narrative synthesis but were not included in pooled estimates unless sufficient comparative data were available.

### GRADE assessment

The certainty of evidence for key outcomes was assessed using the Grading of Recommendations Assessment, Development and Evaluation (GRADE) approach (21). Certainty was rated separately for clinically important outcomes, including pain, opioid consumption, hospital length of stay, respiratory failure, mortality, neuropsychiatric adverse events, sedation, and procedure use. Randomized evidence was initially considered high certainty and observational evidence low certainty, with downgrading based on risk of bias, inconsistency, indirectness, imprecision, and suspected publication bias. Evidence could be rated as high, moderate, low, or very low certainty. Particular attention was given to small sample sizes, wide confidence intervals, clinical heterogeneity in ketamine dose and comparator, active versus placebo comparators, and inclusion of conference abstracts or studies with limited extractable data.

### Publication bias

Publication bias was considered qualitatively. Funnel plots, Begg's test, and Egger's test were not performed because the number of studies contributing to each pooled outcome was small, making these methods unreliable and potentially misleading.

### Statistical analysis

Meta-analysis was performed when at least two studies reported sufficiently comparable outcomes. Quantitative synthesis was performed on an outcome-specific basis. For the purpose of synthesis, comparators were classified as placebo, standard/usual care, or an active analgesic comparator. Pooling required sufficient comparability in outcome definition, measurement scale, assessment period, and availability of numerical data needed to calculate an effect estimate. Active-comparator studies were not pooled with placebo- or usual-care studies because their statistical reporting was incompatible with quantitative synthesis. Continuous outcomes were pooled as mean differences with 95% confidence intervals (CIs) when outcomes were measured on the same scale, including NPS AUC, opioid consumption expressed as MME/OME, and LOS. Dichotomous outcomes were pooled as risk ratios (RR) with 95% CIs; Log RR were used for model estimation and then interpreted as relative effects. Random-effects models were used because of anticipated clinical and methodological heterogeneity across studies, including differences in trauma populations, ketamine dose, route and duration, comparator type, baseline injury severity, analgesic co-interventions, and outcome timing. In case of pooling placebo comparator with standard/usual care comparator, these estimates were interpreted as average effects of a ketamine-based strategy relative to different non-ketamine control strategies rather than as a common effect against an equivalent comparator.

Heterogeneity was assessed using the Cochran Q test, tau-squared, and I^2^ statistics. I^2^ values were interpreted as the proportion of total variation attributable to between-study heterogeneity rather than chance. Prespecified subgroup or subset analyses included opioid outcomes stratified by Injury Severity Score (ISS) when extractable, particularly ISS ≥15 versus ISS <15. Studies with overlapping cohorts were not pooled together for the same outcome.

Additional subgroup analyses or meta-regression according to clinical setting, ketamine dose, route, infusion duration, comparator type, or background multimodal analgesia were planned only if sufficient studies were available within each category. These analyses were not undertaken because most outcome-specific syntheses included only a few studies, and further stratification would have produced very small and statistically unstable subgroups.

Leave-one-out sensitivity analysis was performed for outcome-specific meta-analyses with four or more contributing studies to assess the influence of individual studies on the pooled estimates. For analyses containing non-randomized evidence, omission of the non-randomized study was also used to evaluate the consistency of the findings when restricted to randomized controlled trials. Formal leave-one-out or design-restricted meta-analysis was not performed for outcomes with fewer than four studies because the resulting estimates would be unstable and difficult to interpret. When exclusion of a non-randomized study left only one randomized trial, no pooled RCT-only estimate was calculated. All tests were two-sided, and statistical significance was considered at *p* < 0.05. Analyses were performed using StataMP 17.

## Results

### Study selection and characteristics of included studies

The database search identified 679 records, including 18 from PubMed, 95 from Web of Science, 497 from Scopus, 58 from Embase, and 11 from Cochrane CENTRAL. Two additional records were identified through citation searching. After the removal of 127 duplicates, 552 records were screened. Of these, 390 reports were excluded, and 162 database-derived reports were assessed for eligibility. One additional report identified through citation searching was also assessed. Reports were excluded (*n* = 152), because they were not relevant, did not include rib fracture or chest injury populations, did not include ketamine as the intervention, or were review articles. Overall, 11 studies (8–13, 22–26) were included in the systematic review, and 7 (8–13, 23) contributed data to at least one quantitative synthesis (Figure 1).

**Figure 1:**
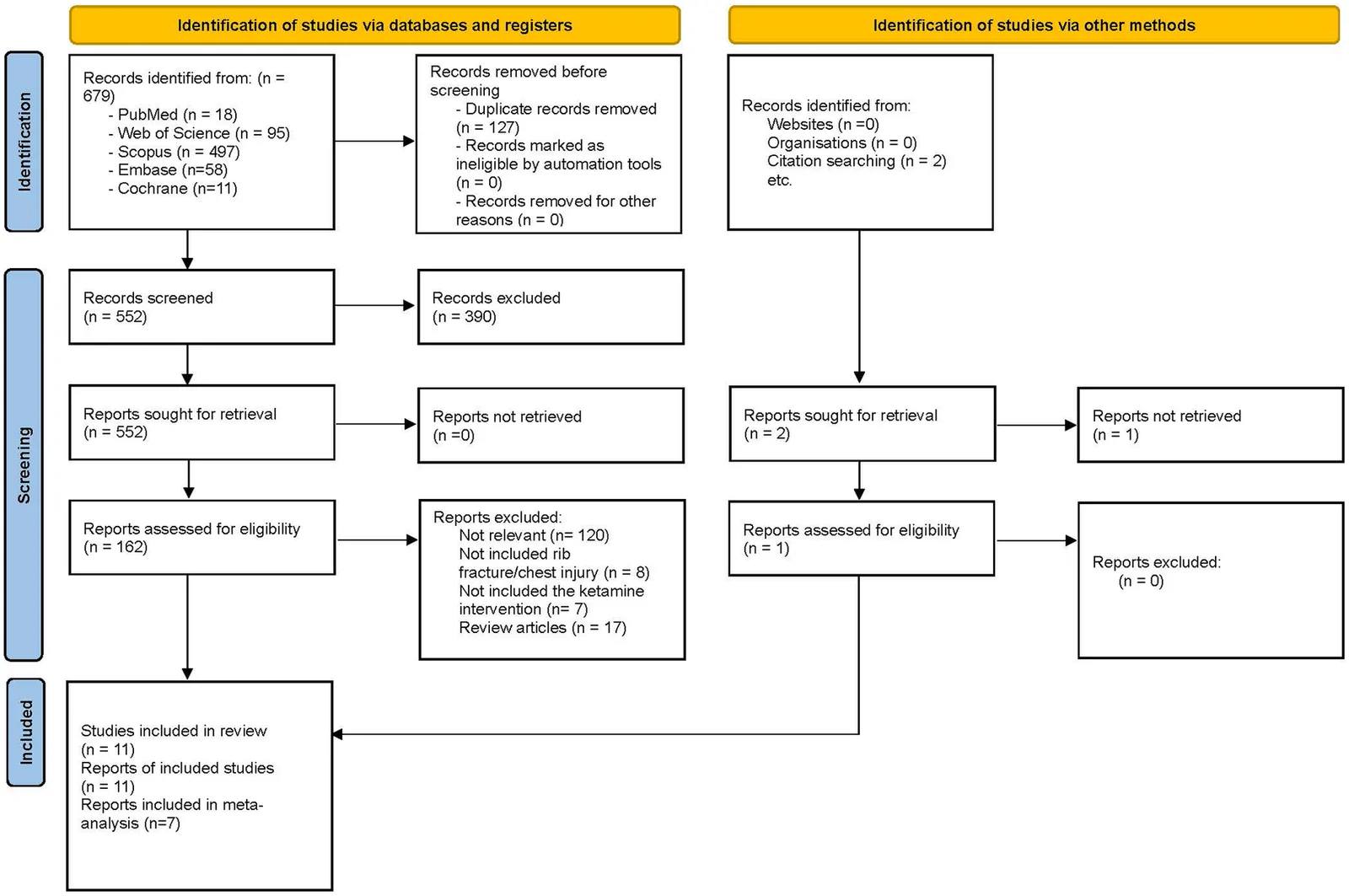
PRISMA flow diagram of study selection.

The included evidence comprised randomized trials, observational comparative evidence, conference abstracts, and uncontrolled descriptive reports. The study populations ranged from adults with multiple traumatic rib fractures to broader traumatic chest injury cohorts. Ketamine was administered either as a continuous low-dose infusion, a titratable infusion during noninvasive ventilation, or a single intravenous analgesic dose. Comparators included placebo or saline infusion, usual multimodal care, ketorolac, lidocaine infusion, or no concurrent control. Study characteristics are presented in Table 1 in detail.

**Table 1:** Characteristics of studies evaluating ketamine for analgesia/sedation in traumatic chest trauma/rib fractures.

Study	Setting and design	Population	Ketamine intervention	Comparator	Sample size in each group	Outcomes reported
Kaban 2015 (26)	USA; Jacobi Medical Center. Conference abstract; retrospective single-arm registry.	Adults >17 years with >=3 blunt-trauma rib fractures receiving ketamine-based pain management.	Low-dose peripheral IV ketamine infusion 0.05 mg/kg/h as part of multimodal analgesia.	No concurrent control.	122 ketamine; no control.	Analgesic co-interventions and qualitative safety/pulmonary complications.
Losing 2016 (22)	USA; Sanford Health. Case series.	Three adult trauma patients with rib fractures and complex associated injuries.	Ketamine infusion titrated 1–10 microg/kg/min.	No control.	3 cases.	Case-level pain and daily MME patterns; adverse event.
Walters 2018 (8)	USA; North Memorial Health Hospital. Retrospective matched case-control cohort.	Adult ICU trauma patients with >=1 rib fracture and ISS >15.	Continuous IV ketamine 0.1 mg/kg/h adjunct to standard care.	Matched standard-care controls without ketamine.	15 ketamine; 15 control.	NPS/BPS, morphine-equivalent use, LOS, mortality, vital signs, hallucination/agitation.
Carver 2019 (9)	USA; Froedtert/Medical College of Wisconsin Level I trauma center. Prospective randomized double-blind placebo-controlled trial.	Adults with >=3 rib fractures; key exclusions included older age, reduced GCS, chronic opioid/ketamine use, and contraindications.	Low-dose ketamine infusion 2.5 microg/kg/min (∼0.15 mg/kg/h) for 48 h plus multimodal rib-fracture protocol.	0.9% saline placebo infusion plus the same protocol.	45 ketamine; 46 placebo.	NPS AUC, OME, ISS > 15 subgroup OME, LOS, epidural, respiratory failure, sedation, hallucinations.
Kugler 2019 (10)	USA; Froedtert/Medical College of Wisconsin Level I trauma center. Prospective randomized double-blind placebo-controlled trial in elderly patients.	Older adults with >=3 rib fractures.	Low-dose ketamine infusion 2 microg/kg/min (∼0.12 mg/kg/h) for 48 h plus multimodal rib-fracture protocol.	0.9% saline placebo infusion plus the same protocol.	30 ketamine; 29 placebo.	NPS AUC, OME as medians/ranges, ISS > 15 subgroup, epidural, respiratory failure, sedation, hallucinations/delirium.
Juul 2019 (25)	USA; conference abstract; *post hoc* discharge-prescribing analysis from the Carver parent trial.	Adult patients with >=3 rib fractures from the Carver randomized cohort.	48-h low-dose ketamine infusion 2.5 microg/kg/min.	Normal saline placebo.	45 ketamine; 46 placebo.	Discharge OME/prescribing outcomes.
Macheel 2024 (11)	USA; North Memorial Health/University of Minnesota. Prospective randomized double-blind placebo-controlled trial.	Hospitalized adults with >1 rib fracture managed under an evidence-based rib-fracture protocol.	Ketamine infusion 0.1 mg/kg/h for up to 48 h plus protocolized multimodal analgesia.	Placebo saline plus the same protocol.	25 ketamine; 25 placebo.	MME day 1/day 2/total, LOS mean(range), pneumonia, respiratory insufficiency/failure.
Ghazaly 2024 (12)	Egypt; Aswan University surgical ICU. Randomized double-blind placebo-controlled three-arm trial.	Adults 18–60 requiring NIV after blunt chest trauma with >3 rib fractures, pulmonary contusion, hypoxemia/hypercapnia, or RR >=25 despite IV analgesia.	IV ketamine 0.20 mg/kg/h during NIV, titrated q60 min by 0.2 mg/kg/h to max 0.5 mg/kg/h to maintain RASS 0 to −3; rescue morphine if VAS >3.	0.9% sodium chloride placebo infusion; dexmedetomidine was a third active arm.	15 ketamine; 15 placebo; 45 total including dexmedetomidine.	NIV duration, VAS, RASS, total morphine, PaO2/FiO2, secondary respiratory complications/chest infection.
Mahmoodabadi 2025 (13)	Iran; three ED hospitals. Randomized double-blind clinical trial.	Adults with chest trauma with fractures of at least two ribs or chest-tube indications.	Single-dose IV ketamine 0.25 mg/kg after IV acetaminophen; rescue morphine as needed.	Single-dose IV ketorolac 30 mg after IV acetaminophen.	45 ketamine; 45 ketorolac.	NRS at baseline/30/60 min, rescue morphine, nausea, nystagmus/visual disturbance.
Klugh 2025 (23)	USA; academic level I trauma center. Pragmatic randomized clinical trial in general trauma.	Adult trauma patients admitted to intermediate/ICU settings; only ∼44–48% had rib fractures.	Bolus 0.35 mg/kg then infusion 0.2 mg/kg/h, titratable 0.1–0.25 mg/kg/h up to 72 h, plus MMPR.	Usual multimodal pain regimen without assigned ketamine.	144 ketamine; 156 usual care.	DVPRS, MME/day, total MME, opioid at discharge, LOS, ICU/ventilator days, mortality, delirium.
Hoyte 2026 (24)	USA; adult Level 1 trauma center. Conference abstract; prospective randomized comparative study.	Adults with >=3 traumatic rib fractures requiring admission.	Ketamine infusion adjunct plus standard multimodal therapy; dose not reported in abstract.	Lidocaine infusion adjunct plus standard multimodal therapy; dose not reported in abstract.	31 ketamine; 30 lidocaine.	OME at 24/48/72 h without dispersion; adverse events.

BPS, Behavioral Pain Scale; ED, emergency department; GCS, Glasgow Coma Scale; ICU, intensive care unit; ISS, Injury Severity Score; LOS, length of stay; MME, morphine milligram equivalent; MMPR, multimodal pain regimen; NPS/NRS, numeric pain scale/rating scale; OME, oral morphine equivalent; RCT, randomized controlled trial; NIV, noninvasive ventilation; PaO2/FiO2, partial pressure of arterial oxygen/fraction of inspired oxygen; RASS, Richmond Agitation-Sedation Scale; VAS, Visual Analog Scale.

The comparator composition differed across the quantitative analyses. The pooled NPS AUC analysis included only the placebo-controlled trials by Carver et al. and Kugler et al. Day-2 opioid consumption, total opioid consumption, the ISS-stratified opioid analyses, epidural placement, respiratory failure, and sedation were likewise synthesized from placebo-controlled comparisons. Hospital length of stay and mortality were analyzed using studies with standard or usual care comparators. Day-1 opioid consumption and hallucinations/delirium/agitation were the only outcome-specific analyses that combined placebo-controlled studies with standard/usual-care comparisons.

The active-comparator studies were not included in the pooled estimates. Mahmoodabadi et al. compared a single intravenous ketamine dose with ketorolac and assessed NRS outcomes at 30 and 60 min, which were not comparable with the NPS AUC outcomes reported in the placebo-controlled infusion trials. Hoyte et al. compared ketamine with lidocaine infusion but reported opioid-equivalent outcomes without sufficient measures of dispersion for quantitative pooling. These studies were therefore included only in the narrative synthesis.

### Quality assessment

Risk of bias was assessed using design-appropriate tools. Randomized trials were assessed using RoB 2. The retrospective matched comparative study was assessed using ROBINS-I. Uncontrolled case series, single-arm abstracts, *post hoc* conference abstracts, and abstract-only comparative studies were appraised separately because conventional comparative risk-of-bias tools are not fully applicable to these designs.

Overall, the randomized placebo-controlled trials generally had better methodological quality than the observational and abstract-only evidence. Ghazaly et al. study (12) was judged at low risk of bias overall, while Carver et al., Kugler et al, Macheel et al., Mahmoodabadi et al., and Klugh et al. studies were judged as having some concerns, mainly due to issues such as small sample size, imbalance in baseline injury severity, pragmatic open-label design, indirectness of the population, or limitations in outcome measurement (9–11, 13, 23). Hoyte et al. was judged as high risk/reporting limitation because it was available only as a conference abstract with missing dispersion data and incomplete methodological detail (24).

Walters et al. study was judged to have serious overall risk of bias using ROBINS-I. Although matching was performed on ISS and prior opioid exposure, the retrospective design, clinician-selected ketamine administration, and potential residual confounding limited causal interpretation (8). Kaban et al., Losing et al., and Juul et al. reports were not considered robust comparative evidence because they were uncontrolled, *post hoc*, or abstract-only reports with limited extractable outcome data (22, 25, 26).

The detailed risk-of-bias tables are provided as supplementary Tables S3–S5 in the supplementary file.

## Quantitative synthesis

### Pain outcomes

Pain outcomes were pooled using the NPS AUC at 12–24 h and 24–48 h from two randomized placebo-controlled rib-fracture trials. Ketamine was not associated with a significant reduction in pain score at 12–24 h (mean difference [MD], −0.10 points; 95% CI, −0.69 to 0.50; I^2^ = 0.0%; *p* = 0.75) or at 24–48 h (MD, 0.24 points; 95% CI, −0.64 to 1.12; I^2^ = 56.5%; *p* = 0.59). The overall pooled estimate across pain time points was also not significant (MD, 0.07 points; 95% CI, −0.40 to 0.54; I^2^ = 21.7%; *p* = 0.77) (Figure 2).

**Figure 2:**
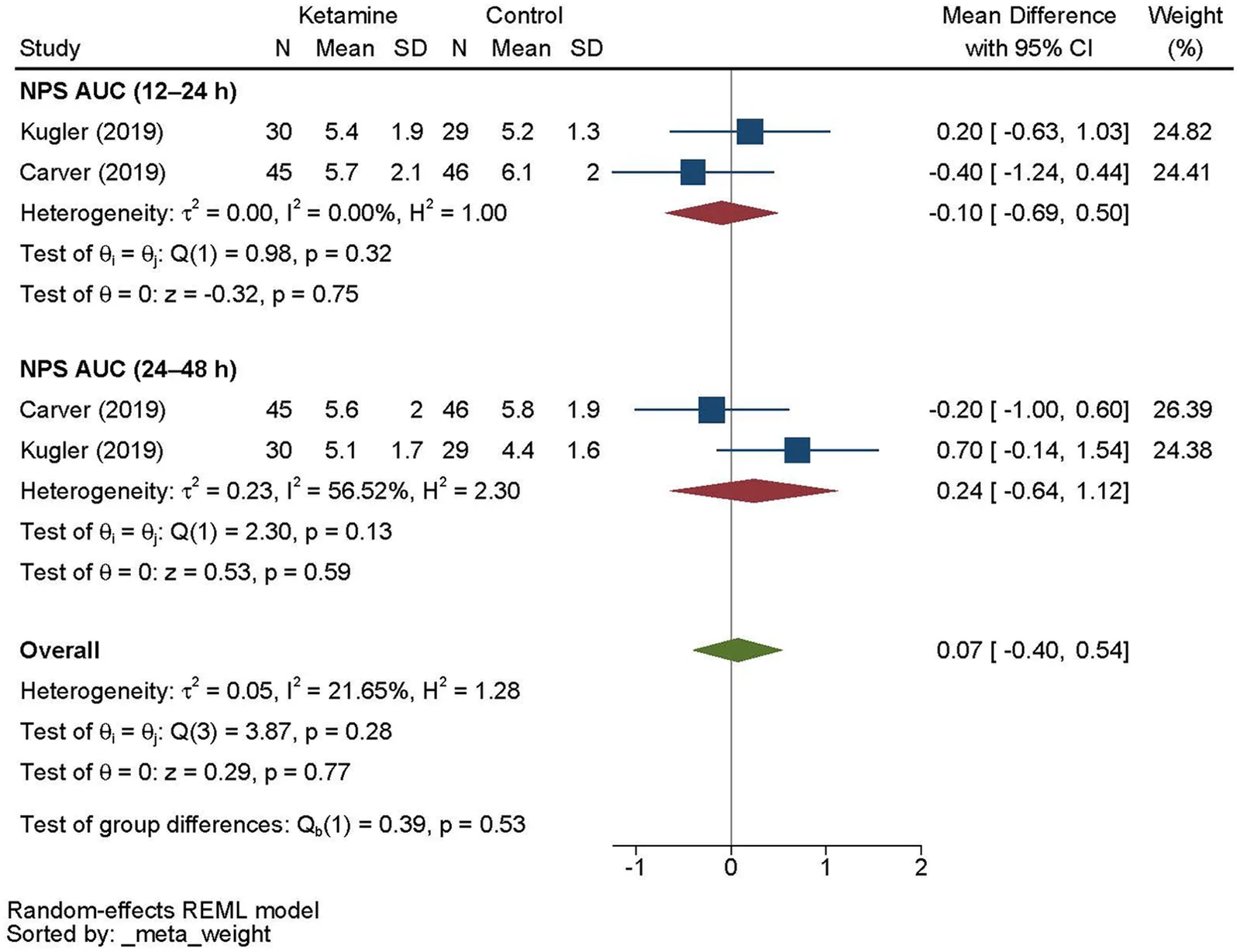
Forest plot of pain outcomes assessed using the numeric pain score area under the curve.

### Opioid consumption

Pooled opioid outcomes were reported as OME/MME according to the original study reporting. For day 1 opioid consumption, ketamine showed a non-significant opioid-sparing trend (MD, −9.55 OME/MME; 95% CI, −20.61 to 1.52; I^2^ = 63.3%; *p* = 0.09). No significant reduction was observed for day 2 opioid use (MD, 9.00 OME/MME; 95% CI, −4.77 to 22.76; I^2^ = 0.0%; *p* = 0.20) or total opioid consumption (MD, −9.35 OME/MME; 95% CI, −25.42 to 6.73; I^2^ = 24.2%; *p* = 0.25). The overall pooled opioid estimate across time strata did not reach statistical significance (MD, −6.82 OME/MME; 95% CI, −15.55 to 1.92; I^2^ = 69.3%; *p* = 0.13) (Figure 3).

**Figure 3:**
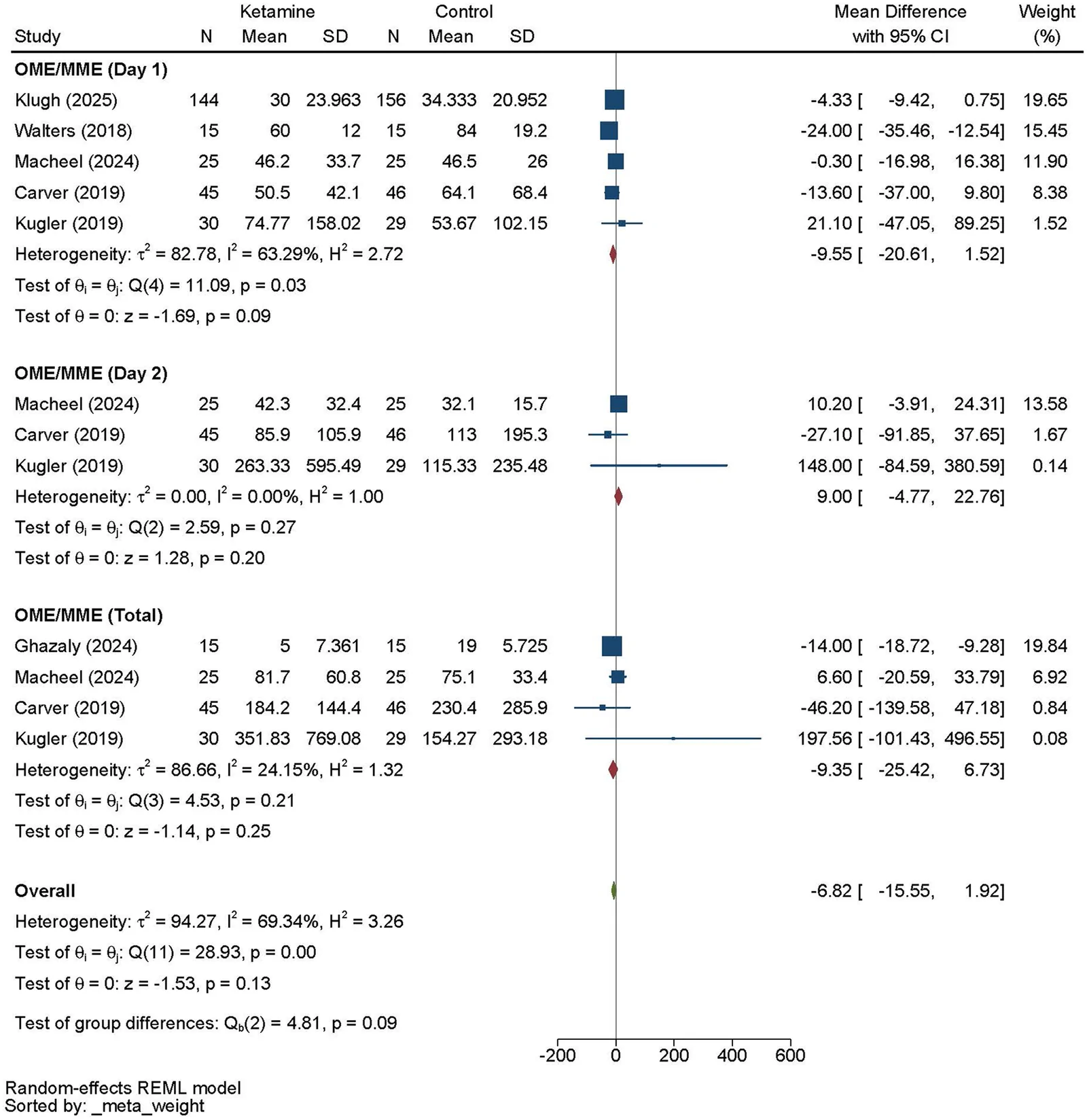
Forest plot of opioid consumption.

In the ISS subgroup analysis, ketamine was associated with lower opioid use among patients with ISS ≥15 during the 12–24-hour interval (MD, −37.39 mg; 95% CI, −73.98 to −0.81; I^2^ = 3.3%; *p* = 0.05). However, this signal was not sustained at 24–48 h (MD, −43.35 mg; 95% CI, −197.64 to 110.95; *p* = 0.58) or for total opioid consumption in the ISS ≥15 subgroup (MD, −72.84 mg; 95% CI, −337.34 to 191.67; *p* = 0.59). In patients with ISS <15, ketamine was not associated with reduced opioid use at 12–24 h, 24–48 h, or overall. The test for subgroup differences was not significant (*p* = 0.28) (Figure 4). Given that this analysis included only two trials with 69 participants in the ISS ≥15 subgroup and showed no statistically significant interaction between ISS categories, the observed 12–24-hour difference was considered exploratory rather than evidence of a differential treatment effect according to injury severity.

**Figure 4:**
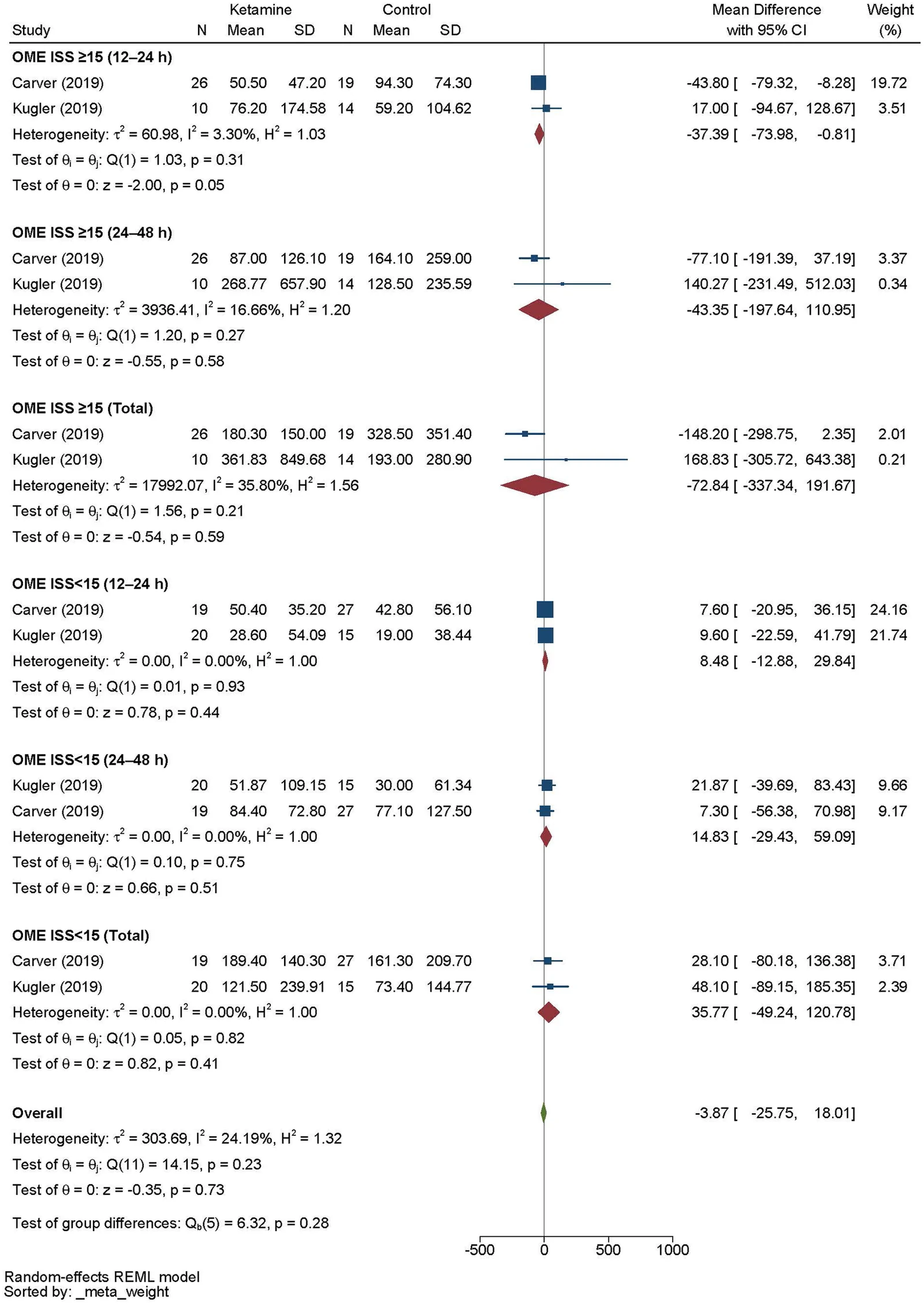
Subgroup forest plot of opioid consumption according to injury severity score.

### Hospital length of stay and procedural outcomes

Hospital length of stay was pooled from two studies and showed no significant difference between the ketamine and control groups (MD, −0.54 days; 95% CI, −5.96 to 4.88; I^2^ = 42.8%; *p* = 0.84) (Figure 5A).

**Figure 5:**
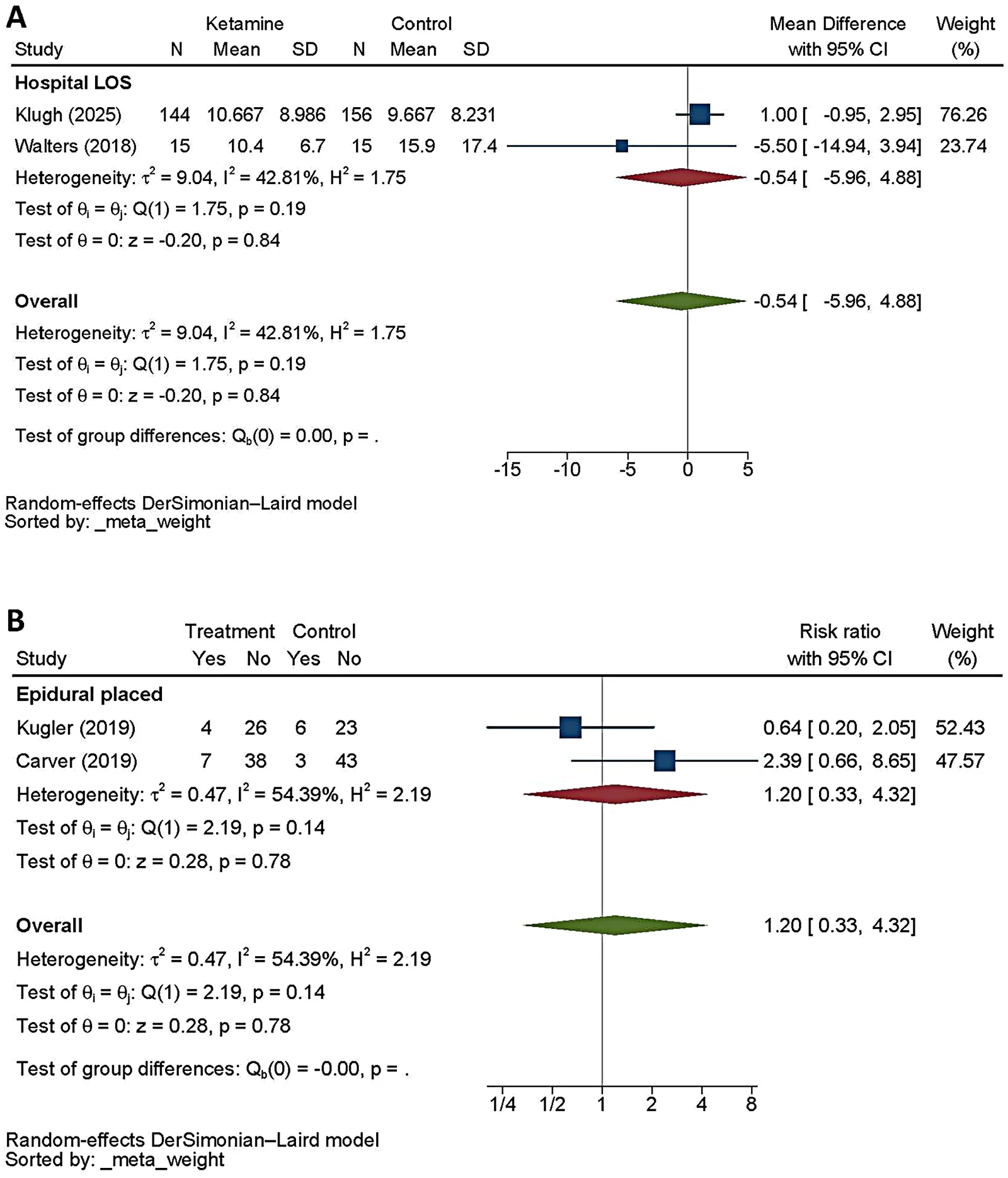
Forest plots of **(A)** hospital length of stay and **(B)** epidural placement.

Epidural placement was reported in two randomized placebo-controlled rib-fracture trials. Ketamine was not associated with a significant difference in epidural placement (risk ratio [RR], 1.20; 95% CI, 0.33 to 4.32; I^2^ = 54.4%; *p* = 0.78). Given the small number of events and wide confidence interval, this estimate should be interpreted cautiously (Figure 5B).

### Safety outcomes

Pooled safety outcomes did not show statistically significant differences between the ketamine and control groups. For hallucinations, delirium, or agitation, the pooled estimate suggested a numerically higher rate with ketamine, but the CI crossed the null (RR, 1.66; 95% CI, 0.83 to 3.29; I^2^ = 0.0%; *p* = 0.15). Mortality was not significantly different (RR, 0.46; 95% CI, 0.09 to 2.45; I^2^ = 31.1%; *p* = 0.36). Respiratory failure was also not significantly different (RR, 0.79; 95% CI, 0.20 to 3.09; I^2^ = 0.0%; *p* = 0.73). Sedation was numerically lower in ketamine-treated patients but not statistically significant (RR, 0.34; 95% CI, 0.07 to 1.67; I^2^ = 0.0%; *p* = 0.18). Across all pooled safety outcomes, the overall estimate did not show a statistically significant difference (RR, 1.03; 95% CI, 0.61 to 1.75; I^2^ = 0.0%; *p* = 0.90) (Figure 6). However, adverse events were sparse, outcome definitions and ascertainment varied, and the confidence intervals included potentially clinically important benefit and harm. These findings therefore should not be interpreted as demonstrating safety or equivalence between ketamine and control intervention.

**Figure 6:**
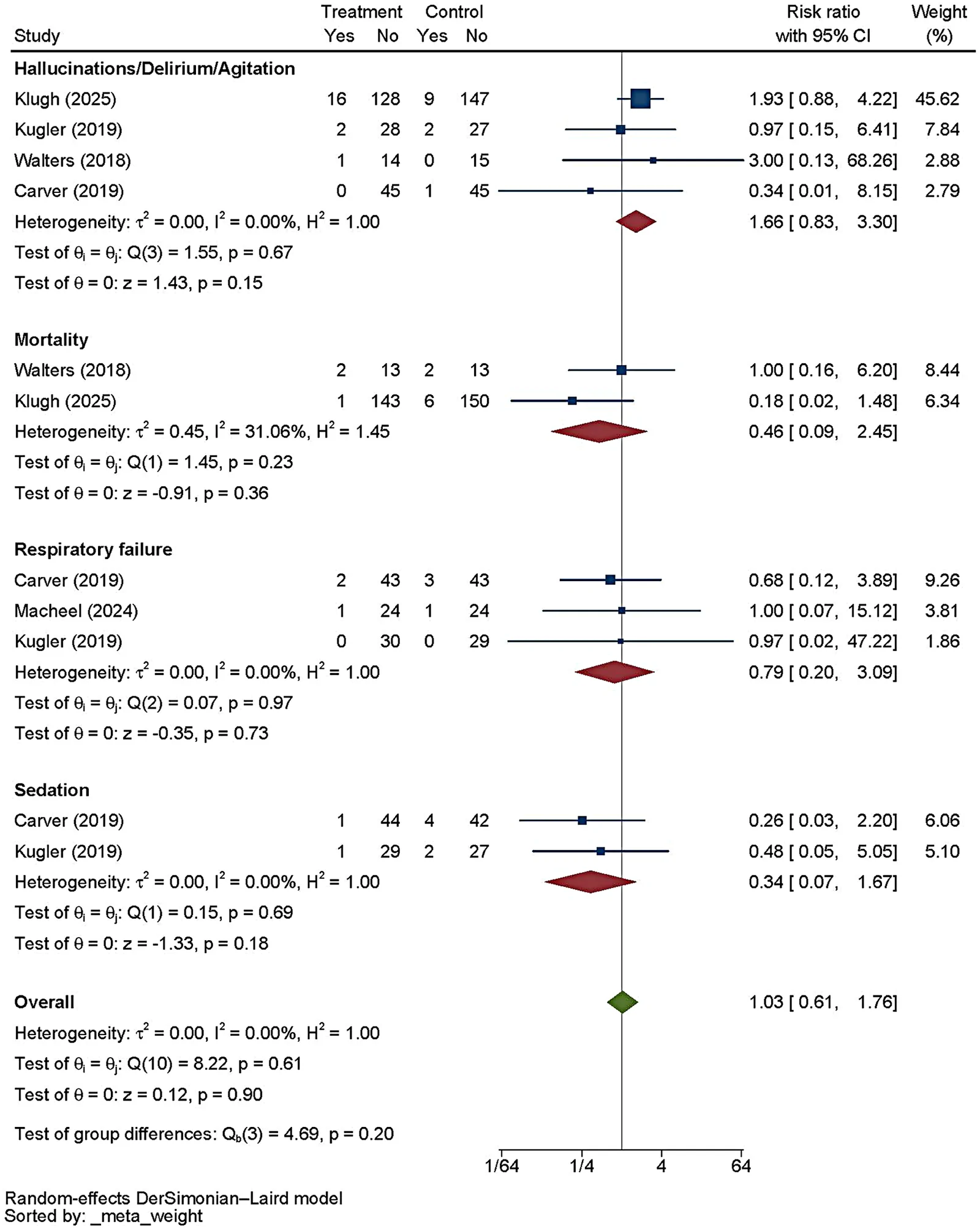
Forest plot of safety outcomes including hallucinations/delirium/agitation, mortality, respiratory failure, and sedation.

### Sensitivity analysis and publication bias

Most outcome-specific meta-analyses were already based exclusively on randomized controlled trials. Walters et al. was the only non-randomized comparative study included in the quantitative synthesis and contributed to the pooled analyses of day-1 opioid consumption, hallucinations/delirium/agitation, hospital length of stay, and mortality. Formal leave-one-out analyses were performed for day-1 opioid consumption and hallucinations/delirium/agitation because these outcomes included at least four studies. For day-1 opioid consumption, omission of Walters et al. attenuated the pooled mean difference, but the result remained non-significant (MD, −4.26 morphine-equivalent mg; 95% CI, −9.01 to 0.49; *p* = 0.078). For hallucinations/delirium/agitation, omission of Walters et al. yielded a pooled log risk ratio of 0.47 (95% CI, −0.23 to 1.18; *p* = 0.189), corresponding approximately to an RR of 1.60 (95% CI, 0.79 to 3.25). Thus, exclusion of the non-randomized study did not materially alter the statistical interpretation of either outcome (Supplementary Figure S1). Hospital length of stay and mortality each included only two studies. Exclusion of Walters et al. would have left only one randomized trial for each outcome; therefore, an RCT-only pooled analysis was not performed. None of the conference abstracts contributed directly to the pooled estimates and they were only included in the narrative synthesis. Formal assessment of publication bias using funnel plots, Begg's test, or Egger's test was not performed because too few studies contributed to each outcome, making these methods unreliable.

### Certainty of evidence

Using GRADE, the certainty of evidence was generally low or very low across outcomes. Evidence for pain outcomes was rated low because the estimates were derived from a few trials and confidence intervals crossed clinically meaningful benefit and no effect. Evidence for overall opioid consumption was low to very low because of inconsistency, indirectness across study populations and comparators, and imprecision. The early opioid-sparing signal in ISS ≥15 patients was rated low because it was based on a small exploratory subgroup. Evidence for hospital length of stay and mortality was rated very low, while evidence for respiratory failure, sedation, and neuropsychiatric adverse events was low because events were sparse and estimates were imprecise. The detailed GRADE table is provided as Supplementary Table S6 in the supplementary file.

## Discussion

In this systematic review and meta-analysis, ketamine did not significantly improve pooled pain scores, total opioid consumption, hospital length of stay, epidural placement, respiratory failure, mortality, sedation, or hallucination/delirium/agitation outcomes among patients with traumatic chest injury and/or rib fractures. The overall evidence therefore, does not support routine ketamine use for all patients with rib-fracture-related pain. However, the findings should not be interpreted as evidence of no possible role for ketamine. Rather, the available data suggest that any benefit is likely context-dependent, influenced by injury severity, background multimodal analgesia, ketamine dose, comparator choice, and outcome timing.

Pain control after traumatic rib fractures is clinically important because inadequate analgesia can impair coughing, deep breathing, mobilization, and pulmonary hygiene, thereby increasing the risk of atelectasis, pneumonia, respiratory deterioration, and prolonged hospitalization (1, 2). In this context, ketamine is an attractive adjunct because subanesthetic dosing may reduce central sensitization and opioid requirements while generally preserving respiratory drive (7, 8). Nevertheless, our pooled pain analysis did not show significant improvement in NPS AUC at 12–24 or 24–48 h. However, the results should be interpreted with caution, as available evidence is limited.

The opioid-sparing results were more nuanced. Overall, opioid use showed a non-significant trend favoring ketamine on day 1, but no significant benefit for day 2 or total opioid consumption. This aligns with the mixed direction of the primary literature. Walters et al. reported lower opioid use in a small matched ICU cohort, and Losing et al. described reduced opioid requirements in selected cases, but these uncontrolled or observational designs are vulnerable to confounding by indication, co-interventions, and clinician selection (8, 22). In contrast, Macheel et al. found no benefit from adding low-dose ketamine to an evidence-based rib-fracture protocol, suggesting that ketamine's incremental value may be limited when high-quality multimodal care is already standardized (11).

An exploratory subgroup analysis suggested lower opioid consumption at 12–24 h among patients with ISS ≥15. However, this finding was based on only two randomized trials involving 69 participants, was not sustained at 24–48 h or for total opioid consumption, and was accompanied by a non-significant test for interaction between ISS subgroups. The available data therefore do not establish that injury severity modifies the analgesic or opioid-sparing effect of ketamine. Although patients with more severe injuries may theoretically have higher baseline opioid requirements and greater potential to benefit from an opioid-sparing adjunct, this clinical rationale should not be interpreted as confirmation of subgroup efficacy. The observed association is best regarded as exploratory and hypothesis-generating and requires prospective confirmation in an adequately powered trial specifically designed or stratified to evaluate treatment effects according to injury severity.

The available sensitivity analyses suggest that the principal statistical interpretations were not driven solely by the non-randomized Walters study. When Walters et al. was omitted from the day-1 opioid-consumption analysis, the apparent opioid-sparing effect was attenuated but remained statistically non-significant. Similarly, exclusion of this study from the hallucinations analysis produced little change in the direction or interpretation of the pooled estimate. These results provide some support for the consistency of the findings when restricted to randomized evidence. However, the sensitivity analyses should be interpreted cautiously. They were feasible for only two outcomes because formal leave-one-out analysis was limited to meta-analyses with at least four studies. For hospital length of stay and mortality, removal of the observational study left only one randomized trial and therefore precluded an RCT-only pooled estimate. The exclusion of conference abstracts had no numerical effect because abstract-only reports contributed only to the narrative synthesis and were not included in any pooled estimate.

The available safety evidence was inconclusive. Ketamine was not associated with a statistically significant increase in hallucinations, delirium, agitation, sedation, respiratory failure, or mortality. However, confidence intervals were wide, and adverse-event definitions varied across studies. Interpretation was further limited by variation in adverse-event definitions and ascertainment. The timing and intensity of surveillance were not standardized, and adverse events may have been influenced by age, traumatic brain injury, ICU admission, concomitant opioids or sedatives, ketamine dose, and treatment duration. These differences may have resulted in under-ascertainment or inconsistent classification of adverse events. Neuropsychiatric outcomes are especially important in older adults, patients with traumatic brain injury, ICU patients, and those receiving sedatives or high-dose opioids. Mahmoodabadi et al. also reported transient nystagmus and nausea with single-dose ketamine, emphasizing that “safety” should not be interpreted only as absence of respiratory depression or mortality (13). Larger trials with standardized adverse-event reporting are needed before firm conclusions can be drawn. However, the available evidence identified no statistically significant safety signal; given the sparse events, heterogeneous outcome definitions, and wide confidence intervals, it remains insufficient to establish the safety or equivalence of ketamine compared with placebo or usual care.

The clinical and methodological heterogeneity of the included studies is central to the interpretation of the pooled estimates. The study populations ranged from adults with multiple traumatic rib fractures treated under protocolized ward-based care to older patients, severely injured ICU cohorts, patients receiving noninvasive ventilation for pulmonary contusion and respiratory compromise, and broader trauma populations in which only a proportion of participants had rib fractures. Baseline pain burden, injury severity, opioid exposure, respiratory vulnerability, and the opportunity to demonstrate an opioid-sparing effect therefore differed substantially across studies. For example, severely injured ICU patients with high baseline analgesic requirements may have greater potential to demonstrate an absolute reduction in opioid consumption, whereas inclusion of less severely injured or heterogeneous general trauma populations may dilute an effect specifically related to rib-fracture pain. Consequently, an average pooled estimate may obscure benefit or harm confined to a more narrowly defined clinical subgroup.

Ketamine exposure also varied from a single intravenous dose to fixed low-dose infusions, titrated infusions during noninvasive ventilation, and bolus-plus-infusion regimens continued for up to 72 h. These regimens are unlikely to be pharmacologically or clinically equivalent, and differences in treatment initiation and outcome-assessment timing could obscure a short-lived early effect or dilute an effect restricted to a particular exposure window. Comparator and co-intervention differences create additional indirectness. Placebo-controlled trials conducted within standardized multimodal rib-fracture protocols estimate the incremental benefit of adding ketamine to otherwise optimized care, whereas comparisons with ketorolac or lidocaine evaluate relative effectiveness against an active analgesic strategy. Usual-care comparisons may additionally be influenced by variation in clinician selection and background treatment. Concomitant acetaminophen, opioid administration, rescue morphine, regional or epidural procedures, sedation strategies, and pulmonary care could reduce the detectable between-group contrast or complicate attribution of efficacy and adverse events specifically to ketamine. Accordingly, the random-effects estimates should be interpreted as average effects across related but not interchangeable clinical contexts rather than as evidence that ketamine has a uniform effect in all patients with traumatic chest injury. These sources of heterogeneity contributed to downgrading the certainty of evidence for inconsistency and indirectness. The findings are most directly applicable to hospitalized adults with multiple traumatic rib fractures receiving subanesthetic intravenous ketamine as an adjunct to multimodal analgesia, whereas applicability to isolated minor injuries, noninvasive-ventilation-specific sedation, emergency-department single-dose treatment, and heterogeneous general trauma populations remains uncertain.

Publication and selective reporting biases remain important sources of uncertainty. The absence of formal funnel-plot or regression-based assessment should not be interpreted as evidence that publication bias was absent, because the small number of studies contributing to each outcome provides insufficient power to identify asymmetry. Small studies with null, unfavorable, or inconclusive findings may have remained unpublished, and their omission could overestimate the efficacy or apparent safety of ketamine. Conversely, because the direction and magnitude of missing evidence are unknown, the influence of publication bias cannot be quantified from the available data. Selective reporting within the included studies may also have affected the synthesis. Studies differed in the outcomes and assessment periods reported, and some provided selected subgroup findings, incomplete adverse-event definitions, or outcome values without sufficient measures of dispersion. This may lead to preferential inclusion of more favorable or more readily extractable results and may particularly limit interpretation of safety outcomes, for which infrequent events and non-standardized reporting could result in under-ascertainment. Several randomized studies were judged to have some concerns regarding outcome measurement or selection of the reported result, while the abstract-only evidence had more substantial limitations in completeness and methodological reporting. Conference abstracts were included to avoid excluding potentially relevant grey literature and emerging evidence; however, their inclusion presents a trade-off. Abstracts may reduce the risk of omitting studies that have not reached full publication, but they provide limited information regarding prespecified outcomes, analytic decisions, missing data, adverse-event ascertainment, and risk of bias. In the present review, the three abstract-only reports were included exclusively in the narrative synthesis and did not directly influence the pooled estimates. Their findings were therefore treated as preliminary and were not used to support firm efficacy or safety conclusions. Overall, the possible influence of unpublished studies and selective reporting reinforces the need for cautious interpretation of the low- to very-low-certainty evidence.

## Strengths and limitations

This review has several strengths. It focused on a clinically specific and practical question, separated narrative and quantitative synthesis when data were not poolable, evaluated multiple clinically relevant domains, and incorporated pain, opioid-sparing, procedural, length-of-stay, and safety outcomes. However, the limitations are important. First, most pooled outcomes included only two to five studies, frequently with small sample sizes or sparse events, resulting in limited statistical power, imprecision, and low or very low certainty of evidence. Consequently, non-significant findings may reflect insufficient power rather than the absence of a clinically meaningful benefit or harm. Estimates of statistical heterogeneity were also imprecise because few studies contributed to each analysis; therefore, a low I^2^ value should not be interpreted as definitive evidence of clinical homogeneity. Safety outcomes were particularly limited by sparse event counts, inconsistent definitions, non-standardized ascertainment, and wide confidence intervals.

Second, study populations were heterogeneous with respect to age, injury severity, number of rib fractures, ICU admission, pulmonary contusion, noninvasive ventilation, and concomitant injuries. Third, ketamine dosing varied substantially, from single-dose Emergency Department administration to fixed low-dose and titratable infusion protocols with different treatment durations.

Fourth, comparator interventions and background multimodal analgesia protocols differed across studies. Most quantitative analyses were restricted to either placebo-controlled or standard/usual-care comparisons, although placebo and standard/usual care were combined for day-1 opioid consumption and hallucinations/delirium/agitation. The ketorolac- and lidocaine-controlled studies were included only in the narrative synthesis and did not directly influence the pooled estimates. Some trials evaluated ketamine as an adjunct to a standardized multimodal rib-fracture protocol applied similarly to both groups, whereas usual-care studies allowed greater variation in systemic opioid administration, non-opioid analgesics, rescue medications, regional analgesia, and other co-interventions. These differences may attenuate or amplify the incremental effect attributable to ketamine and limit the applicability of the pooled estimates to any single institutional analgesic protocol. Because few studies were available within each comparator category, reliable comparator-stratified subgroup analyses or meta-regression could not be performed.

Fifth, outcome definitions, measurement methods, and assessment periods were inconsistent. Pain was assessed using different instruments and summary measures, opioid consumption was reported using different morphine-equivalent metrics and time windows, and adverse events were variably defined and ascertained. Importantly, using different methods for opioid equivalence calculation may contribute to measurement heterogeneity despite harmonization; therefore, residual measurement heterogeneity may remain even after harmonization of reporting periods. These differences may limit the clinical interpretability of the pooled estimates. Although random-effects models were used to account for between-study variability, statistical modeling cannot fully resolve clinically meaningful differences in population, intervention, comparator, co-interventions, and outcome timing. Moreover, the small number of studies contributing to most outcomes precluded reliable subgroup analyses or meta-regression according to clinical setting, injury severity, ketamine dose, route, infusion duration, comparator type, or background multimodal analgesia.

Sixth, the observational evidence remained susceptible to confounding by indication and residual confounding. Although Walters et al. matched participants according to Injury Severity Score and prior opioid exposure, ketamine administration was clinician-selected, and unmeasured differences in baseline pain severity, injury complexity, anticipated opioid requirements, eligibility for alternative analgesic strategies, concomitant treatments, or clinical trajectory may have influenced both treatment allocation and outcomes. Therefore, associations observed in this study should not be interpreted as causal treatment effects.

Seventh, three reports were available only as conference abstracts. Although their inclusion reduced the possibility of completely excluding grey or emerging evidence, incomplete methodological reporting, absent or insufficient dispersion data, and uncertainty regarding selective outcome reporting limited their interpretability and risk-of-bias assessment. These reports were included only in the narrative synthesis and did not directly contribute to the principal pooled estimates. Additionally, publication bias could not be formally evaluated because too few studies contributed to each outcome. Consequently, unpublished small studies or selectively reported null, unfavorable, or safety findings cannot be excluded. Finally, several outcomes important to patients and clinicians, such as pulmonary complications, ventilator days, opioid prescribing at discharge, chronic pain, and functional recovery, were sparsely reported in the available original studies.

## Future directions and unresolved questions

The available evidence does not identify a patient subgroup that has been conclusively shown to derive greater benefit from ketamine. In current practice, ketamine may nevertheless be considered on an individualized basis as a monitored adjunct to multimodal analgesia in hospitalized adults with painful multiple rib fractures or blunt chest trauma when pain remains inadequately controlled despite conventional treatment, when reducing opioid exposure is a particular clinical priority, or when other systemic or regional analgesic options are contraindicated, unavailable, or insufficient. This potential role should be viewed as selective and context-dependent rather than as support for routine ketamine use. In particular, the apparent early opioid-sparing signal among patients with ISS ≥15 remains exploratory, was derived from a small subgroup, and does not establish that injury severity predicts treatment benefit. It also remains uncertain whether ketamine is more useful in older adults, ICU patients, patients with pulmonary contusion or noninvasive ventilation requirements, individuals with high baseline opioid exposure, or those unable to receive regional analgesia. Other unresolved questions include the optimal dose, bolus versus infusion strategy, timing and duration of treatment, interaction with the background multimodal analgesic protocol, comparative effectiveness against active alternatives such as NSAIDs, lidocaine, or regional techniques, and effects on pulmonary complications, ventilator duration, functional recovery, chronic pain, discharge opioid prescribing, and uncommon adverse events.

Future investigations should prioritize adequately powered, multicenter randomized trials in clearly defined rib-fracture and blunt chest-trauma populations. Trials should prospectively stratify or randomize according to clinically relevant characteristics, including injury severity, age, number of rib fractures, pulmonary contusion, ICU admission, baseline opioid exposure, and eligibility for regional analgesia. The exploratory opioid-sparing finding in patients with ISS ≥15 requires prospective confirmation through an adequately powered interaction analysis. Future studies should directly compare standardized ketamine regimens, including bolus, fixed low-dose infusion, and titrated infusion strategies, within clearly defined multimodal analgesic protocols. Pain outcomes, opioid-equivalent calculations, pulmonary endpoints, neuropsychiatric and respiratory adverse events, discharge opioid prescribing, functional recovery, and longer-term pain outcomes should be defined prospectively and reported consistently.

## Conclusion

In this systematic review and meta-analysis, ketamine did not significantly improve pooled pain scores, overall opioid consumption, hospital length of stay, epidural placement, respiratory failure, mortality, sedation, or neuropsychiatric adverse events among patients with traumatic chest injury and/or rib fractures. Although the overall findings do not support routine ketamine administration for patients with rib-fracture-related pain, an exploratory reduction in opioid consumption at 12–24 h was observed in a small subgroup of patients with ISS ≥15. This finding was based on only two trials, was not sustained at later time points, and was not supported by a statistically significant subgroup interaction. It should therefore be regarded as hypothesis-generating rather than evidence that patients with greater injury severity derive a differential benefit from ketamine. No statistically significant differences were detected in the evaluated safety outcomes; however, adverse events were infrequent, definitions and ascertainment methods varied, and confidence intervals were wide, and the findings should be interpreted cautiously.

Overall, the certainty of evidence remains low to very low because of small sample sizes, heterogeneous populations and treatment protocols, sparse adverse events, inconsistent safety definitions, and imprecise pooled estimates. Current evidence is insufficient to identify a subgroup that clearly derives greater benefit from ketamine. Its use as part of multimodal analgesia should therefore remain individualized and should not be preferentially recommended on the basis of ISS alone. Future adequately powered, multicenter randomized trials with prespecified injury-severity strata, standardized dosing, outcome definitions, opioid-equivalent reporting, and adverse-event monitoring are needed to determine whether any clinically relevant subgroup benefits from ketamine.

## Data Availability

The original contributions presented in the study are included in the article/Supplementary Material, further inquiries can be directed to the corresponding author.
