# Supplementary material for: Safety, efficacy, and opioid-sparing effects of ketamine for analgesia in traumatic chest injury and rib fractures: a systematic review and meta-analysis
Source: Front Med (Lausanne). 2026 Sep 29;13:1914190. doi: 10.3389/fmed.2026.1914190 (PMC13658463; doi:10.3389/fmed.2026.1914190)
Supplement: Supplementary file 1 [file Supplementaryfile1.docx]

**Supplementary file**

**Table S1- Keywords used for search in different databases**

| Rib Fracture*  Chest Injur*  Thoracic Injur* | Ketamine  2-(2-Chlorophenyl)-2 (methylamino)cyclohexanone  Ketalar  CI 581  Ketanest  Ketamine Hydrochloride  Calipsol  Calypsol  Kalipsol  Ketaset |
| --- | --- |

**Table S2- Detailed search strings used in each database**

| **Database** | **Search string** | **Results** |
| --- | --- | --- |
| **PubMed** | #1  (((((((((Ketamine[Title/Abstract]) OR (2-(2-Chlorophenyl)-2-(methylamino)cyclohexanone[Title/Abstract])) OR (Ketalar[Title/Abstract])) OR (CI 581[Title/Abstract])) OR (Ketanest[Title/Abstract])) OR (Ketamine Hydrochloride[Title/Abstract])) OR (Calipsol[Title/Abstract])) OR (Calypsol[Title/Abstract])) OR (Kalipsol[Title/Abstract])) OR (Ketaset[Title/Abstract]) | [26,475](https://pubmed.ncbi.nlm.nih.gov/?term=%28%28%28%28%28%28%28%28%28Ketamine%5BTitle%2FAbstract%5D%29+OR+%282-%282-Chlorophenyl%29-2-%28methylamino%29cyclohexanone%5BTitle%2FAbstract%5D%29%29+OR+%28Ketalar%5BTitle%2FAbstract%5D%29%29+OR+%28CI+581%5BTitle%2FAbstract%5D%29%29+OR+%28Ketanest%5BTitle%2FAbstract%5D%29%29+OR+%28Ketamine+Hydrochloride%5BTitle%2FAbstract%5D%29%29+OR+%28Calipsol%5BTitle%2FAbstract%5D%29%29+OR+%28Calypsol%5BTitle%2FAbstract%5D%29%29+OR+%28Kalipsol%5BTitle%2FAbstract%5D%29%29+OR+%28Ketaset%5BTitle%2FAbstract%5D%29&sort=) |
|  | #2  ((Rib Fracture*[Title/Abstract]) OR (Chest Injur*[Title/Abstract])) OR (Thoracic Injur*[Title/Abstract]) | [9,420](https://pubmed.ncbi.nlm.nih.gov/?term=%28%28Rib+Fracture%2A%5BTitle%2FAbstract%5D%29+OR+%28Chest+Injur%2A%5BTitle%2FAbstract%5D%29%29+OR+%28Thoracic+Injur%2A%5BTitle%2FAbstract%5D%29&sort=) |
|  | #1 AND #2 | [18](https://pubmed.ncbi.nlm.nih.gov/?term=%28%28%28%28%28%28%28%28%28%28Ketamine%5BTitle%2FAbstract%5D%29+OR+%282-%282-Chlorophenyl%29-2-%28methylamino%29cyclohexanone%5BTitle%2FAbstract%5D%29%29+OR+%28Ketalar%5BTitle%2FAbstract%5D%29%29+OR+%28CI+581%5BTitle%2FAbstract%5D%29%29+OR+%28Ketanest%5BTitle%2FAbstract%5D%29%29+OR+%28Ketamine+Hydrochloride%5BTitle%2FAbstract%5D%29%29+OR+%28Calipsol%5BTitle%2FAbstract%5D%29%29+OR+%28Calypsol%5BTitle%2FAbstract%5D%29%29+OR+%28Kalipsol%5BTitle%2FAbstract%5D%29%29+OR+%28Ketaset%5BTitle%2FAbstract%5D%29%29+AND+%28%28%28Rib+Fracture%2A%5BTitle%2FAbstract%5D%29+OR+%28Chest+Injur%2A%5BTitle%2FAbstract%5D%29%29+OR+%28Thoracic+Injur%2A%5BTitle%2FAbstract%5D%29%29&sort=) |
| **Web of Science** | #3  Ketamine (Topic) or 2-(2-Chlorophenyl)-2-(methylamino)cyclohexanone (Topic) or Ketalar (Topic) or CI 581 (Topic) or Ketanest (Topic) or Ketamine Hydrochloride (Topic) or Calipsol (Topic) or Calypsol (Topic) or Kalipsol (Topic) or Ketaset (Topic) | [36,874](https://www.webofscience.com/wos/woscc/summary/31ad9cc9-fda7-4fe9-8a55-e34f713283e7-01b0e1e85c/relevance/1) |
|  | #4  Rib Fracture* (Topic) or Chest Injur* (Topic) or Thoracic Injur* (Topic) | [45,234](https://www.webofscience.com/wos/woscc/summary/1477de8e-b78d-4fec-9cda-652e02285a37-01b0e1e302/relevance/1) |
|  | #3 AND #4 | [95](https://www.webofscience.com/wos/woscc/summary/57e6c918-7341-4b1f-b26c-d139100754f3-01b0e1ea1f/relevance/1) |
| **Scopus** | #5  ( TITLE-ABS-KEY ( Ketamine ) OR TITLE-ABS-KEY ( 2 ( 2-Chlorophenyl ) 2 ( methylamino ) cyclohexanone ) OR TITLE-ABS-KEY ( Ketalar ) OR TITLE-ABS-KEY ( CI 581 ) OR TITLE-ABS-KEY ( Ketanest ) OR TITLE-ABS-KEY ( Ketamine Hydrochloride ) OR TITLE-ABS-KEY ( Calipsol ) OR TITLE-ABS-KEY ( Calypsol ) OR TITLE-ABS-KEY ( Kalipsol ) OR TITLE-ABS-KEY ( Ketaset ) ) | 81,828 results |
|  | #6  ( TITLE-ABS-KEY ( Rib Fracture* ) OR TITLE-ABS-KEY ( Chest Injur* ) OR TITLE-ABS-KEY ( Thoracic Injur* ) ) | 92,571 results |
|  | #5 AND #6 | 497 results |
| Embase | #7  ('ketamine' OR '2-(2-chlorophenyl)-2-(methylamino)cyclohexanone' OR 'ketalar' OR 'CI 581' OR 'ketanest' OR 'ketamine hydrochloride' OR 'calipsol' OR 'calypsol' OR 'kalipsol' OR 'ketaset'):ti,ab,kw | 41,611 results |
|  | #8  ('rib fracture*' OR 'chest injur*' OR 'thoracic injur*'):ti,ab,kw | 13,180 |
|  | #7 AND #8 | 58 results |
| Cochrane | #9  (ketamine OR "2-(2-chlorophenyl)-2-(methylamino)cyclohexanone" OR ketalar OR "CI 581" OR ketanest OR "ketamine hydrochloride" OR calipsol OR calypsol OR kalipsol OR ketaset) :ti,ab,kw | 9246 |
|  | #10 ("rib fracture*" OR "chest injur*" OR "thoracic injur*") :ti,ab,kw | 369 |
|  | #9 AND #10 | 11 |

# **Table S3. Risk of bias in randomized and randomized-comparative studies using Rob2 tool**

| **Study** | **Design** | **Randomization or confounding** | **Deviations from intended interventions** | **Missing outcome data** | **Outcome measurement** | **Selection of reported result / reporting** | **Overall judgment** |
| --- | --- | --- | --- | --- | --- | --- | --- |
| Carver 2019 | Parallel double-blind placebo-controlled RCT | Low | Low | Low | Some concerns | Some concerns | Some concerns |
| Kugler 2019 | Parallel double-blind placebo-controlled RCT in elderly patients | Low | Low | Low | Some concerns | Some concerns | Some concerns |
| Macheel 2024 | Parallel double-blind placebo-controlled RCT | Some concerns | Some concerns | Low | Low | Low | Some concerns |
| Ghazaly 2024 | Three-arm double-blind placebo-controlled RCT | Low | Low | Low | Low | Low | Low |
| Mahmoodabadi 2025 | Double-blind active-comparator RCT | Low | Some concerns | Low | Some concerns | Low | Some concerns |
| Klugh 2025 | Pragmatic randomized comparative-effectiveness trial | Low | Some concerns | Low | Low | Low | Some concerns |
| Chapman 2026 | Conference abstract; prospective RCT | Some concerns | Some concerns | Not assessable | Some concerns | Some concerns | High |

Low = low risk/limitation; Some concernse = possible limitations that may affect confidence; High = important limitations; Not assessable = insufficient reporting or not applicable.

# **Table S4. Risk of bias in non-randomized comparative study using ROBINS-I**

| **Study** | **Design** | **Confounding** | **Selection of participants** | **Intervention classification** | **Deviations / co-interventions** | **Missing data** | **Outcome measurement** | **Selective reporting** | **Overall judgment** |
| --- | --- | --- | --- | --- | --- | --- | --- | --- | --- |
| Walters 2018 | Retrospective matched case-control cohort | Serious | Moderate | Low | Moderate | Low | Moderate | Moderate | Serious |

Low = low risk/limitation; Moderate = possible limitations that may affect confidence; Serious = important limitations.

# **Table S5. Appraisal of uncontrolled, post hoc, and abstract-only reports using JBI appraisal tool**

| **Study** | **Design / tool** | **Patient selection / representativeness** | **Exposure and outcome ascertainment** | **Completeness of reporting** | **Comparability / control of confounding** | **Overall judgment** |
| --- | --- | --- | --- | --- | --- | --- |
| Kaban 2015 | Conference abstract; retrospective single-arm registry review | Some concerns | Some concerns | High | High | High |
| Losing 2016 | Three-patient case series | High | Some concerns | Some concerns | High | High |
| Juul 2019 | Conference abstract; post hoc discharge-prescribing analysis from Carver parent RCT | Some concerns | Some concerns | High | Some concerns | High |

Some concerns= possible limitations that may affect confidence; High= important limitations

**Table S6. GRADE evidence profile for key outcomes**

| **Outcome** | **Studies / participants** | **Pooled effect** | **Heterogeneity** | **Certainty of evidence** | **Downgrading rationale** |
| --- | --- | --- | --- | --- | --- |
| Pain, NPS AUC 12-24 h | 2 RCTs / 150 participants | MD -0.10 points (95% CI -0.69 to 0.50) | I2=0.0% | Low | Downgraded for risk of bias/some concerns in outcome measurement and imprecision; confidence interval crosses no effect and clinically relevant thresholds. |
| Pain, NPS AUC 24-48 h | 2 RCTs / 150 participants | MD 0.24 points (95% CI -0.64 to 1.12) | I2=56.5% | Low | Downgraded for some risk-of-bias concerns, inconsistency, and imprecision. |
| Opioid consumption, Day 1 | 5 studies / 530 participants | MD -9.55 MME/OME (95% CI -20.61 to 1.52) | I2=63.3% | Very low | Downgraded for risk of bias, indirectness from mixed populations/comparators, inconsistency, and imprecision. |
| Opioid consumption, Day 2 | 3 RCTs / 200 participants | MD 9.00 MME/OME (95% CI -4.77 to 22.76) | I2=0.0% | Low | Downgraded for some risk-of-bias concerns and imprecision. |
| Total opioid consumption | 4 RCTs / 230 participants | MD -9.35 MME/OME (95% CI -25.42 to 6.73) | I2=24.2% | Low | Downgraded for risk-of-bias concerns, indirectness in outcome timing/population, and imprecision. |
| ISS >=15 subgroup, OME 12-24 h | 2 RCTs / 69 participants | MD -37.39 mg (95% CI -73.98 to -0.81) | I2=3.3% | Low | Downgraded for subgroup/exploratory nature and imprecision from small sample size. |
| ISS >=15 subgroup, total OME | 2 RCTs / 69 participants | MD -72.84 mg (95% CI -337.34 to 191.67) | I2=35.8% | Very low | Downgraded for subgroup nature, imprecision, and inconsistency. |
| Hospital length of stay | 2 studies / 330 participants | MD -0.54 days (95% CI -5.96 to 4.88) | I2=42.8% | Very low | Downgraded for inclusion of one observational study, indirectness, inconsistency, and imprecision. |
| Epidural placement | 2 RCTs / 150 participants | RR 1.20 (95% CI 0.33 to 4.31) | I2=54.4% | Low | Downgraded for imprecision and inconsistency. |
| Respiratory failure | 3 RCTs / 200 participants | RR 0.79 (95% CI 0.20 to 3.10) | I2=0.0% | Low | Downgraded for very few events and imprecision. |
| Hallucinations/delirium/agitation | 4 studies / 480 participants | RR 1.65 (95% CI 0.83 to 3.29) | I2=0.0% | Low | Downgraded for risk of bias/indirectness and imprecision. |
| Sedation | 2 RCTs / 150 participants | RR 0.34 (95% CI 0.07 to 1.67) | I2=0.0% | Low | Downgraded for very few events and imprecision. |
| Mortality | 2 studies / 330 participants | RR 0.46 (95% CI 0.09 to 2.46) | I2=31.1% | Very low | Downgraded for indirectness, risk of bias, and very serious imprecision due to few events. |

Abbreviations: AUC, area under the curve; CI, confidence interval; GRADE, Grading of Recommendations Assessment, Development and Evaluation; ISS, Injury Severity Score; MD, mean difference; MME, morphine milligram equivalents; NIV, noninvasive ventilation; NPS, numeric pain score; OME, oral morphine equivalents; RCT, randomized controlled trial; ROBINS-I, Risk Of Bias In Non-randomized Studies of Interventions; RoB 2, revised Cochrane risk-of-bias tool for randomized trials; RR, risk ratio.

**A**

**B**

**Supplementary Figure S1. Leave-one-out sensitivity analyses for (A) OME/MME day 1 and (B) hallucinations/delirium/agitation.**
